# An Effective Photocatalytic Degradation of Industrial Pollutants through Converting Titanium Oxide to Magnetic Nanotubes and Hollow Nanorods by Kirkendall Effect

**DOI:** 10.3390/nano12030440

**Published:** 2022-01-27

**Authors:** Osama Saber, Hicham Mahfoz Kotb, Mostafa Osama, Hassan A. Khater

**Affiliations:** 1 Department of Physics, College of Science, King Faisal University, P.O. Box 400, Al-Ahsa 31982, Saudi Arabia; hkotb@kfu.edu.sa (H.M.K.); 214110595@student.kfu.edu.sa (M.O.); hkhater@kfu.edu.sa (H.A.K.); 2 Petroleum Refining Department, Egyptian Petroleum Research Institute, P.O. Box 11727, Nasr City 11765, Egypt; 3 Physics Department, Faculty of Science, Assiut University, Assiut 71516, Egypt

**Keywords:** kirkendall effect, magnetic nanotubes, hollow nanorods, photocatalytic degradation of dyes, effective removal of dyes

## Abstract

Controlling of morphology from nanoparticles to magnetic nanotubes and hollow nanorods are interesting for developing the photo-active materials and their applications in the field of photocatalysis and decontamination of aquatic effluents. In the current study, titanium dioxide nanoparticles and nanocomposites were prepared by different techniques to produce various morphologies. The nanoparticles of pure titanium dioxide were prepared by sol-gel technique. Magnetic nanotubes and hollow nanorods were prepared by combining titanium with di- and tri-valent iron through two stages: urea hydrolysis and solvent thermal technique. According to the Kirkendall effect, magnetic nanotubes were fabricated by unequal diffusion of Fe^2+^, Fe^3+^ and Ti^4+^ inside the nanocomposite to produce maghemite-titanian phase. In the same trend, hollow nanorods were synthesized by limited diffusion of both trivalent iron and tetravalent titanium producing amorphous structure of titanium iron oxides. The magnetic and optical properties showed that these nanotubes and hollow nanorods are magnetically active and optically more effective compared with titanium dioxide nanoparticles. Therefore, the Naphthol green B dye completely disappeared after 45 min of UV light irradiation in presence of the hollow nanorods. The kinetic study confirmed the high performance of the hollow nanorods for the photocatalytic degradation of Naphthol green B compared with titanium dioxide nanoparticles.

## 1. Introduction

Currently, one of the main targets of the scientific communities is creating a sustainable society. It aims at decontamination of aquatic effluents through developing clean and efficient technologies based on renewable resources. In this way, one of the most effective and clean techniques is photocatalysis. The optical energy is used in the photocatalysis processes as a driving force for converting organic pollutants to water and carbon dioxide. However, the high recombination of photo-generated charges and the low visible light absorption limit the ability of the photo-active materials for commercializing this technique in a wide area [1]. Recently, many researchers considered that sunlight is a good solution for cleaning water from the colored pollutants through using semiconductors in photocatalytic degradation processes of dyes [2,3].

Photocatalytic degradation has used oxidation reactions as effective forces for oxidizing and decomposing toxic organic pollutants [4]. Light radiation is a source of energy and photons for activating and exciting photocatalysts to produce oxidizing agents which consider the critical step for these photocatalytic reactions. An external UV light or natural solar light can be used as a source of photons. In these reactions, hydroxyl radicals (OH.), which consider very reactive species, are produced and are in charge of the oxidation of organic pollutants [4]. Among various optical materials, much attention has been given to titanium dioxide TiO_2_ [5]. According to the band gap energy of titanium dioxide, electrons are excited and generated after absorbing 3.3 eV from UV light producing holes inside the valance band of titanium dioxide [6,7]. Widespread environmental applications of titanium dioxide are due to strong oxidizing power in addition to nontoxicity, low cost, resistance to photo-corrosion, chemical and biological inertness [8,9,10]. A wide band gap and fast recombination of electron-hole pairs limited the ability of titanium dioxide in the photocatalytic degradation of pollutants. 

To remove the limitations of TiO_2_ and widen its application, the combined structures between Ti and other species were formed for increasing the efficient charge separation and light harvesting [11,12,13]. Zhang’s research group reported that a high performance in photocatalytic degradation of the dye molecules was achieved by MoS_2_-TiO_2_ nanobelts [14]. Also, a high visible-light photocatalytic activity was observed by MoS_2_ nanosheets/TiO_2_ nanowires [15]. Gao et al., designed a composite photocatalyst based on Graphene/TiO_2_ for improving photocatalysis property [16]. Core–shell hetero-structures of TiO_2_/MoS_2_ were prepared to decrease the band gap energy of titanium dioxide [17,18]. In the same trend, the Fe_3_O_4_/TiO_2_/MoS_2_ hetero-structures were prepared and tested in the decomposition of a rhodamine B solution under visible light by Sun et al. [19]. They reported that these hetero-structures showed highly photocatalytic activities in aqueous solutions and easily separated by using an external magnet. Isik et al., and Padervand et al., have used graphene oxide with titanium dioxide for improving its photocatalytic properties and inhibiting the recombination of electron–hole pairs because the high conductivity and conjugated system of graphene oxide sheets worked as better electron acceptors [20,21]. Rana et al. [22,23] have used different structures of Ni and Fe oxides instead of titanium dioxide as photocatalysts. They reported that the efficiency of photocatalytic degradation of the pollutants increased because of their low band gap. Also, they indicated that these composites were easily collected and separated from the water using a magnet because of their magnetic behavior. Recently, an effective photodegradation of the pesticide was reported by Anirudhan et al. through synthesizing manganic composite consisting of graphene oxide, nickel ferrite and titanium dioxide [24]. 

Many authors have reported that the morphology of the optical materials plays an important role in the optical applications [25,26,27,28,29,30,31,32,33]. Nanotubes and nanorods showed new or enhanced properties compared with the other morphology. The nanotubes of iron oxide showed an excellent photo activity comparing with the nanoparticles [30]. Also, the charge transportability in the nanotubes was approximately 45 times higher than the nanoparticles. In the same trend, the light-harvesting and charge-collection efficiencies were higher in the nanotubes of titanium dioxide than the nanoparticles [33]. 

In the current study, all these advantages can be collected through designing magnetic nanotubes and hollow nanorods by combining titanium dioxide and iron oxide inside a maghemite-titanian structure using Kirkendall effect. 

The Kirkendall effect is a familiar process occurring inside metals and oxides [34,35,36]. It basically depends on a non-mutual diffusion process between two metals through their interface so that vacancies are produced and moved to compensate for the un-equality of the flow of materials. In the nano world, a new fabrication route is produced for designing hollow nano-objects depending on the Kirkendall effect [37,38,39,40].

To the best of our knowledge, few studies were observed in the literature on the preparation of maghemite-titanian nanotubes and hollow nanorods. For this study, hollow nanorods and nanotubes were designed through the Kirkendall process for the first time and their photocatalytic activities were studied through photocatalytic degradation of the industrial dyes in detail. Also, the optical and magnetic properties of both hollow nanorods and nanotubes were studied and compared with the titanium dioxide nanoparticles. 

## 2. Materials and Methods

**Figure 1:**
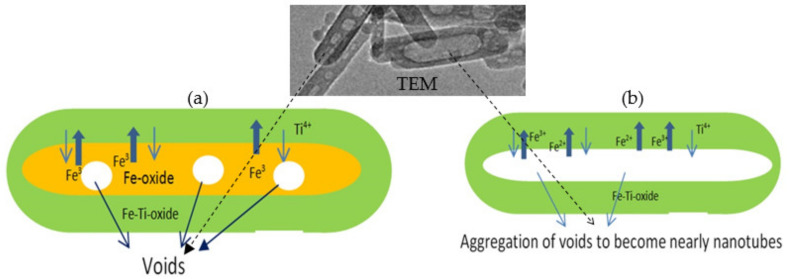
(**a**) Kirkendall-induced hollow nanorods formation and (**b**) Kirkendall-induced nanotubes formation.

In order to indicate the effective role of Kirkendall effect on the morphology and the optical and magnetic properties of titanium dioxide for removing industrial dyes through insertion of different species of iron, nanoparticles of titanium dioxide were prepared by Sol-Gel technique for comparison. In this trend, two different types of titanium iron oxides nanocomposites were prepared by combining urea hydrolysis and solvent thermal technique. The first type of nanocomposites depended on divalent iron. The second one was prepared using trivalent iron. Depending on the different diffusion rate of di- and trivalent ions of iron with tetravalent titanium, the Kirkendall effect has a strong role in producing hollow nanorods and nanotubes, as shown in Figure 1.

### 2.1. Preparation of Titanium Dioxide Nanoparticles

Sol-Gel method was used for preparing the nanoparticles of titanium dioxide. Titanium (IV) isopropoxide was used as a titanium precursor because it is less corrosive and easier to handle. Titanium (IV) isopropoxide (97%) and 2-propanol were obtained from Sigma-Aldrich Company. Typically, 120 mL of titanium (IV) isopropoxide was mixed with 2-propanol to obtain a clear solution. Another aqueous solution was prepared by mixing 70% water and 30% 2-propanol to form a mixed solvent. The prepared mixed solvent was added to the solution of titanium (IV) isopropoxide to produce Sol form. Then, by strong magnetic stirring, the Sol form transformed to Gel form of titanium hydroxide. It means that the hydrolysis of Titanium isopropoxide occurred by water through the consecutive changing of isopropoxide groups (-OC_3_H_7_) with hydroxyl groups (-OH) to form a dispersion of particles of titanium species in liquid, which itself is called Sol form. By condensation and polymerization processes, a Sol form transformed to Gel of titanium hydroxide. A yellow precipitate was collected by using centrifuge at 2500 rpm. After drying at 90 °C, a clear white powder was obtained and coded as TTO sample. In the second stage of preparation, the white powder was thermally treated at 450 °C for converting titanium hydroxide to a crystalline structure of titanium dioxide.

### 2.2. Preparation of Titanium Iron Oxide Nanocomposites

The first type of titanium iron oxide was prepared by two stages. In the first stage, urea hydrolysis was used. This type was produced by combining tetravalent titanium with divalent iron salts. At first, an aqueous solution of divalent iron Fe^2+^ was prepared to obtain 0.1688 M of iron dichloride. Also, another aqueous solution of tetravalent titanium Ti^4+^ was prepared to obtain 0.0302 M of titanium tetrachloride. By magnetic stirring, 500 mL of the first aqueous solution was mixed with the same amount of the second solution to get a clear mixture. 1 mol of urea was added to the mixture. After heating the mixture up to 90 °C for 12 h, a brown precipitate was produced and washed several times with filtration by deionized water. The product was dried in vacuum at room temperature. In the second stage, this product has thermally treated under high pressure by solvent thermal technique. 1.5 g of the prepared powder was dispersed in 350 mL of methyl alcohol by magnetic stirring for 3 h. After that, the mixture was kept inside autoclave. The temperature gradually increased up to 300 °C until the pressure inside the autoclave arrived at 90 bar. By cooling the autoclave in presence of nitrogen, the powder was collected and coded by TF-2.

The second type of titanium iron oxides was prepared by the same procedure with replacement divalent iron Fe^2+^ with trivalent iron Fe^3+^. Two stages have been used to combine tetravalent titanium with trivalent iron salts. In the first stage, iron trichloride reacted with titanium tetrachloride during urea hydrolysis to produce a fine powder. In the second stage, the prepared fine powder was treated inside the autoclave at the same conditions as mentioned in the first type. The product collected and coded by TF-3.

### 2.3. Physical Characterization

Crystalline structures of the prepared materials were determined by X-ray diffraction analysis (XRD) using a Bruker-AXS system (Bruker Company, Karlsruhe, Germany) with Cu-Ka radiation. The different elements in the prepared materials were identified by Energy dispersive X-ray spectroscopy (EDX) through an electron probe micro analyser JED 2300 (JEOL Company, Tokyo, Japan). Thermal analyses of the prepared materials were measured by two devices: differential scanning calorimetry (DSC) TA series Q600 and thermogravimetric analyzer TA series Q500 (TA company, New Castle, PA, USA). Imaging the nano size and morphology of the prepared materials were measured by scanning electron microscopy (SEM) JEOL, JSM-6330F system (15 kV/12 mA) and transmission electron microscopy (TEM) JEM 2100F (JEOL Company, Tokyo, Japan) with an acceleration voltage of 200 kV. 

### 2.4. Optical and Magnetic Properties

In order to determine the optical properties of the prepared materials, the diffuse reflectance technique has been used through UV/VIS/NIR Shimadzu 3600 spectrophotometer (Shimadzu, Columbia, MD, USA) which attached with an integrating sphere ISR-603 for measuring the solid materials. The magnetic properties of the prepared materials were measured through Vibrating Sample Magnetometer (VSM) (Micro Sense, East Lowell, MA, USA). These measurements were carried out at 300 K with vibrating sample magnetometer mode lEV9.

### 2.5. Photocatalytic Activity

The photocatalytic activity of the prepared materials was measured through photocatalytic degradation of industrial dyes. In this trend, quartz immersion well reactor RQ400, which was provided by photochemical reactors limited company in Camberley Surrey, United Kingdoom, was used for performing photocatalytic reactions. This kind of reactor is among the most efficient for photochemical reactions because the lamp is effectively surrounded by the solution to be irradiated. The lamp is contained in double-walled immersion wells made of quartz, allowing water cooling. 400 W medium pressure mercury lamp 3040/PX0686 was used with 400-watt power supply 3140/PX0783. The reactions were achieved inside 400 mL standard reaction flask Model 3308, which is made of quartz glass. In the current study, green dye (Naphthol green B) is used as a pollutant. Aqueous solution of Naphthol green B was prepared with low concentration 4 × 10^−4^ M. According to the law of Beer–Lambert, when the initial concentration of dye is low, the intensity of the measured spectrum of the dye can be used to express the dye concentration. Therefore, the change of the concentration of Naphthol green B is followed by measuring the absorbance at 714 nm, which considers the characteristic peak of Naphthol green B. By using photocatalytic reactor, the green solution was mixed with one of the prepared materials and irradiated by UV light at room temperature. To keep the temperature of the photocatalytic reaction, the glass reactor was equipped with a cooling system. Before irradiation of UV light and after stirring the mixture for 5 min in the dark, the concentration of the green mixture was measured for detecting the adsorption of the dye on the catalyst. At different intervals of irradiation time, a fixed amount of the solution was extracted and measured by UV-Vis spectrophotometer to determine the concentration of the remaining dye in the solution. 

## 3. Results

The prepared titanium dioxide before and after thermal treatment were characterized by X-ray diffraction, thermal analyses and scanning electron microscopy. Also, design of hollow nanorods and nanotubes of titanium iron oxides were identified and analyzed by transmission electron microscopy, X-ray diffraction and thermal analyses. By comparing with the nanoparticles of titanium dioxide, the enhanced optical and magnetic properties of the hollow nanorods and nanotubes led to high activity for removing the Naphthol green B dye from water using light.

### 3.1. Characterization of the Prepared Titanium Dioxide Nanoparticles

To design nanoparticles, the organic and inorganic solvents used in the preparation method have a clear effect on the behavior of the prepared titanium dioxide. In our case, water and 2-propanol were used as solvents to control and obtain small nano-size for titanium dioxide according to the following Equation:

Ti(OC_3_H_7_)_4_ + 2H_2_O + HOC_3_H_7_ = Ti(OC_3_H_7_)_4-n_(OH)_n_ + HOC_3_H_7_ (1)

**Figure 2:**
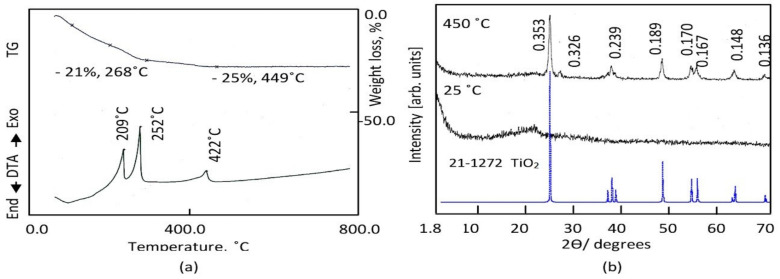
The prepared sample TTO: (**a**) Thermal analyses and (**b**) X-ray diffraction.

Organic solvent can act as a modifier to slow down the hydrolysis reaction of titanium precursor. Figure 2a shows the thermal analyses of the prepared sample TTO through the thermal gravimetric curve and the differential thermal analysis.

TG curve showed that 25 wt.% of the prepared sample TTO was lost with heating up to 449 °C. This gradual weight loss may be due to the removal of organic species. This speculation was confirmed by observing two exothermic peaks at 209 °C and 252 °C in DTA curve agreeing with this weight loss. These exothermic peaks are due to oxidation reactions for organic groups. It means that the prepared sample TTO has titanium dioxide coated by 25% organic species. Also, there is one exothermic peak observed at 422 °C without weight loss indicating crystallization of titanium dioxide. Therefore, the prepared sample TTO was thermally treated at 450 °C in the second stage of preparation method to get crystalline structure of titanium dioxide.

X-ray diffraction patterns of the prepared sample before and after thermal treatment are shown in Figure 2b. Non-crystalline structure was observed for the prepared sample TTO before thermal treatment as seen in Figure 2b. After thermal treatment, the X-ray diffraction pattern showed that the sample TTO-450 has crystalline structure agreeing with the diffraction lines of titanium dioxide. By comparing with the standard diffraction pattern of JCPDS 21-1272, Figure 2b showed that the sample TTO-450 has anatase phase; where the characterized reflections of TTO-450 are observed at 2Ѳ = 25.27, 38.15, 48.04, 54.15, 55.21, 62.95 and 69.11 agreeing with the lattice planes of (101), (004), (200), (105), (211), (204), and (220), respectively. In addition, a weak peak was observed at 2Ѳ = 28.3 as shown in Figure 2b. It is a characteristic peak for a rutile phase according to JCPDS 21-1276. It means that the sample TTO-450 has anatase phase doped with low percentage of rutile phase. Noting that a clear broadness was observed for all diffraction lines of TTO-450, it suggests that the particles size of TTO-450 is in the nano scale. This speculation was confirmed by scanning electron microscopy.

**Figure 3:**
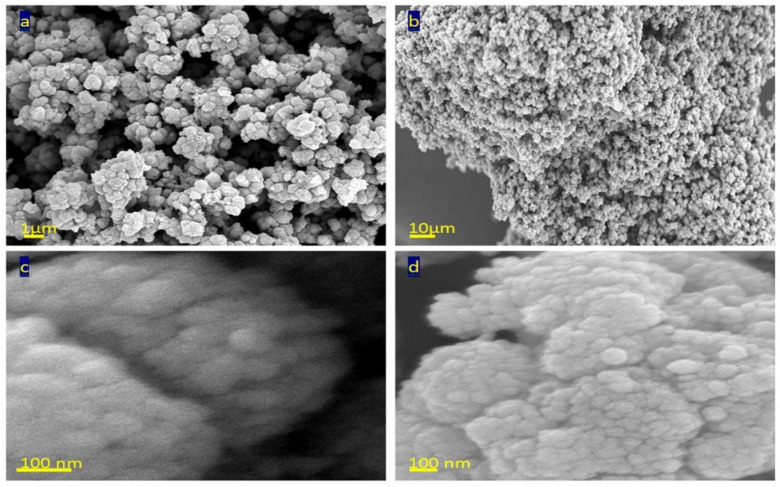
SEM images of TTO-450 by different magnifications: (**a**) 1 µm, (**b**) 10 µm and (**c**,**d**) 100 nm.

Scanning electron microscopy analysis of TTO-450 was displayed in Figure 3 after coating with thin film of platinum to get high resolution images. Figure 3a showed clusters of nanoparticles forming a porous structure. Also, strong aggregation was observed in Figure 3b, indicating strong surface forces among the nanoparticles. By magnification at 100 nm, clear and spherical nanoparticles were observed in Figure 3c,d. The size of the nanoparticles is 50 nm.

### 3.2. A Design of Magnetic Nanotubes

**Figure 4:**
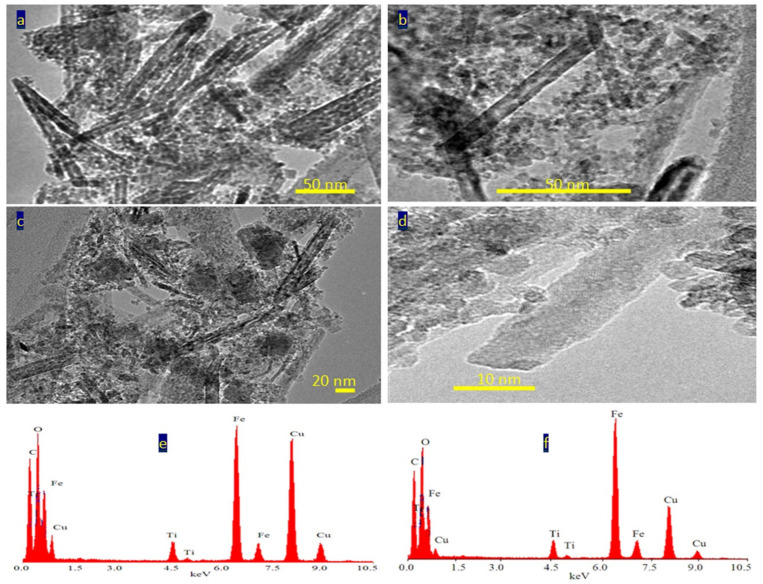
The sample TF-2: (**a**–**d**) TEM images and (**e**,**f**) EDX spectra.

Figure 4 reveals transmission electron microscopy images of the sample TF-2. Nanotubes were observed in Figure 4a. Figure 4b showed individual nanotubes with length less than 100 nm. By magnification, TEM images showed that the diameter of nanotubes is 10 nm, as seen in Figure 4c,d.

The nanotubes morphology of the sample TF-2 could be explained by the Kirkendall effect, as seen later in the Mechanism section [37,38,39,40]. In the beginning, the reaction produced Ti_x_Fe_1-x_ (OH)_2_ in a rod form. According to the partial oxidation of Fe^2+^ and the different diffusion rate of Fe^2+^/Fe^3+^/Ti^4+^ ions inside the nanorods, multiple supersaturated vacancies were produced leading to formation of multiple small voids. These multiple small voids started to aggregate and produce large one void to minimize the surface energy. Because of the extreme Kirkendall effect [37,38] of the ternary system Fe^2+^/Fe^3+^/Ti^4+^, these nanorods have a good chance for forming complete nanotubes as shown in TEM images. The different elements in the sample TF-2 were identified by measuring energy dispersive X-ray spectrometry (EDX) analysis as shown in Figure 4e,f. Figure 4e,f showed the EDX spectrum in two locations in the sample. Titanium, iron and oxygen were observed in the two diagrams indicating homogeneity for the prepared sample. The peaks of copper and carbon are due to TEM-grid, which is usually made of copper with a mesh size of about 3 mm and a thin film of carbon.

**Figure 5:**
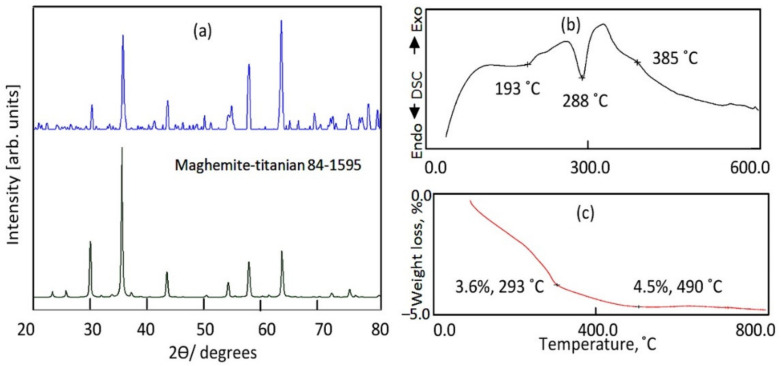
The sample TF-2 (**a**) X-ray diffraction, (**b**) DSC curve and (**c**) TG curve.

The structure of the sample TF-2 was identified by X-ray diffraction as shown in Figure 5a. Figure 5a showed that it has crystalline structure according to the standard card of maghemite-titanian (JCPDS 84-1595). It is a type of titanium iron oxide. The main reflections of the sample TF-2 agree with the planes of maghemite-titanian (220), (311), (400), (422), (511) and (440). It means that the chemical formulae of the sample TF-2 are Fe_0.23_(Fe_1.95_Ti_0.42_)O_4_, agreeing with JCPDS 84-1595. According to the calculation of oxidation states, the iron exists inside the structure as 11% Fe^2+^ and 89% Fe^3+^. 

The thermal stability of this structure was confirmed by the thermal analyses of the sample TF-2 as shown in Figure 5b,c. TG curve showed that only 4.5 wt.% were lost with heating up to 800 °C. By DSC curve, two endothermic peaks were observed at 193 °C and 288 °C agreeing with the weight loss in TG curve. These peaks are due to dehydration and dehydroxylation processes [41].

### 3.3. A Design of Magnetic Hollow Nanorods

**Figure 6:**
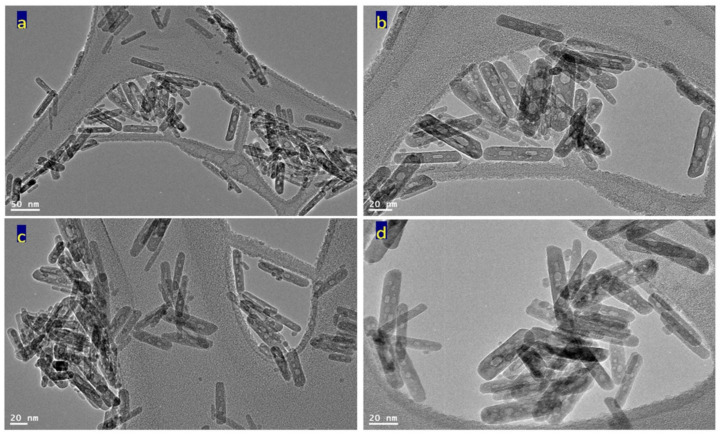
TEM images of the sample TF-3: (**a**) at 50 nm, (**b**) at 20 nm, and (**c**,**d**) other locations.

**Figure 7:**
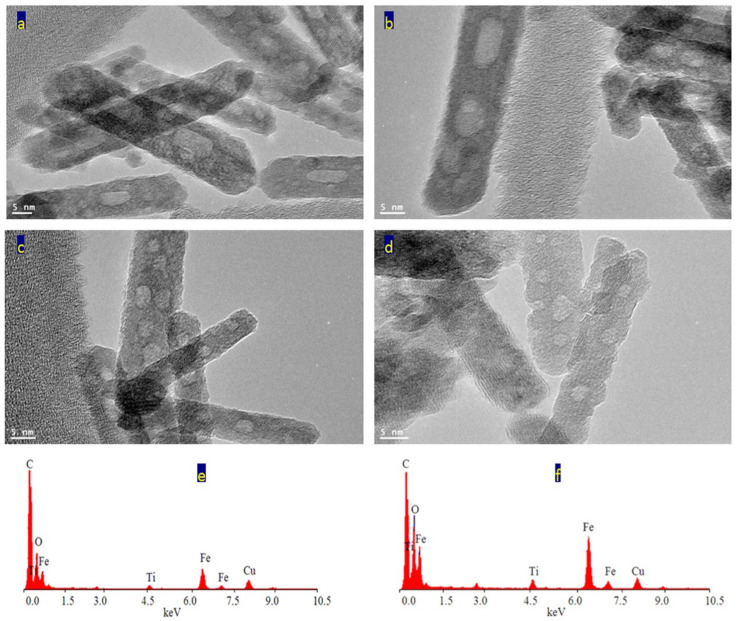
The sample TF-3: (**a**–**d**) TEM images after magnification and (**e**,**f**) EDX spectra.

Transmission electron microscopy images of the prepared sample TF-3 were displayed in Figure 6 and Figure 7. Clear nanorods were observed for the sample TF-3. Figure 6 showed short and individual nanorods. By magnification, Figure 7 showed that these nanorods have 10 nm in width or thickness and 50 nm in length. Also, nano-voids were observed inside these nanorods. Figure 7c showed that the distribution of voids covers a large area of the nanorods. The size of the voids is in the range of 2–5 nm. 

The scenario of void formation could be explained by the Kirkendall effect [37]. This effect depends on an unequal partial diffusion between two different atoms such as titanium and iron in addition to oxygen to produce spinel structure. This diffusion is simultaneously accompanied by creating vacancies. Condensation of excess vacancies can give rise to void formation within the fast-diffusion side [42,43,44]. Accordingly, these vacancies are very likely to get supersaturated and coalesce into a single void. The utilization of the Kirkendall effect in the nano scale became a familiar route for producing hollow nanostructures from a large number of compounds [37].

The analysis of EDX confirmed that the sample TF-3 is composed of three elements as shown in Figure 7e,f. The peaks of iron, titanium and oxygen were observed in one of the nanorods. In addition, the same elements were identified in a very small spot of the nanorod, as seen in Figure 7f. The peaks of both copper and carbon are due to TEM-grid.

**Figure 8:**
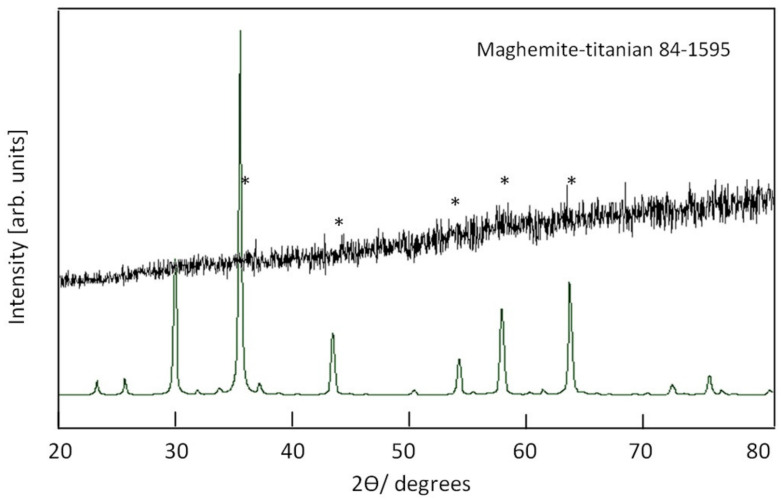
X-ray diffraction of the sample TF-3.

X-ray diffraction pattern of the sample TF-3 is displayed in Figure 8. It shows that the sample TF-3 has a non-crystalline structure. Weak peaks were observed in Figure 8 indicating that the sample TF-3 has a maghemite, titanian structure in an amorphous state.

### 3.4. Mechanism of Hollow Nanorods and Nanotubes

In 2004, Yin et al. [45] reported that hollow nanospheres of CoS were formed through Kirkendall effect during reaction of cobalt nanoparticles with a solution of sulfur. Also, similar results were observed in the same study during formation of CoO. After that, the formation of hollow nanoparticles according to the Kirkendall effect has become very familiar. Many researchers have used this technique for designing a wide range of hollow nanoparticles such as oxides [46], phosphides [47], sulfides [48], metals [49], fluorides [50] and selenides [51].

In the current study, TEM images showed that the formation of both hollow nanorod and nanotubes for titanium iron oxides could be explained by Kirkendall effect. 

**Figure 9:**
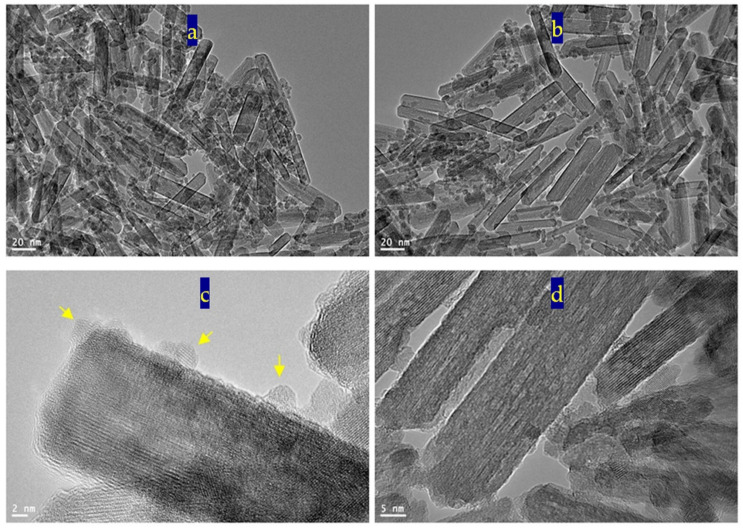
TEM images of TF-3 in the first stage of preparation: (**a**,**b**) two locations, (**c**) at 2 nm and (**d**) at 5 nm.

In the first stage of preparation, Figure 9 showed that titanium iron oxide nanorods were formed and an excess of iron oxide was produced as small nanoparticles. Figure 9a,b indicated that dimensions of the nanorods are 60 nm in length and 10 nm in width. The size of the nanoparticles was less than 5 nm. Figure 9c shows that the nanoparticles of iron oxide tend to diffuse inward through the surface of titanium iron oxide nanorods. Small voids were observed in some nanorods as seen in Figure 9d. 

**Figure 10:**
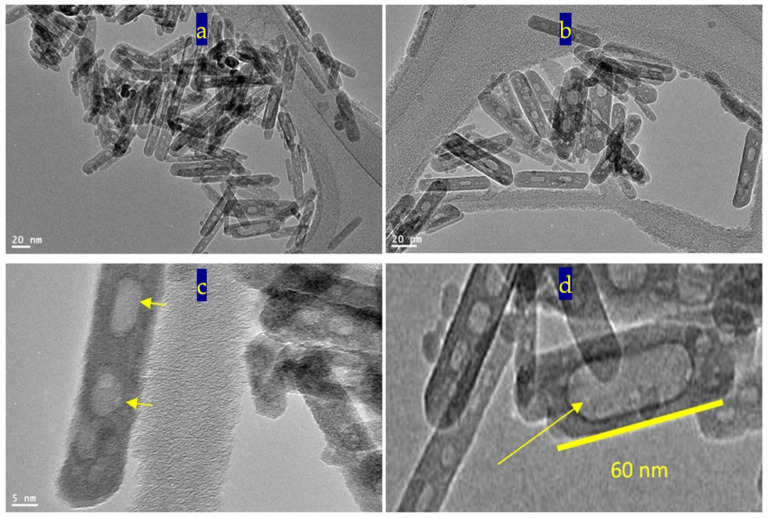
TEM images of TF-3 in the second stage of preparation: (**a**,**b**) two locations, (**c**) at 5 nm and (**d**) at 10 nm.

In the second stage of preparation, after reaction inside the autoclave in presence of high pressure and temperature, the nanoparticles completely disappeared, as shown in Figure 10. It means that the diffusion process of the nanoparticles inward the nanorods were accelerated inside the autoclave. At the same time, large voids were observed in all the nanorods as seen in Figure 10a,b. Figure 10c showed that the voids have irregular shapes with size in the range of 5–10 nm in diameter. According to the Kirkendall effect, the voids were formed because titanium atoms diffused in opposite directions from the core of the nanorods (outward direction). 

According to Kirkendall-type diffusions, the bulk diffusion of the growth species and vacancies are continued and controlled by the Fick’s first law [52]:

J = −D dc(2)

where D is the diffusion coefficient, J is the flux of atoms, and dc is the potential gradient of the atom concentrations. This illustrates that the concentration gradient has an important role for driving the diffusion at the interface. The void widens only by aggregating the inward-flux excess vacancies at the core center. This is clear in Figure 10c,d. The voids are large and concentrated at the middle part of the nanorods. In some nanorods, the size of the void inside the nanorod increased to be 50 nm and became very near to forming a nanotube, as shown in Figure 10d. This finding was confirmed by using divalent iron instead of trivalent iron because all nanorods transformed to nanotubes as shown in Figure 4. In case of using divalent iron, an extreme Kirkendall effect was observed leading to expanding the voids to obtain crystalline spinel nanotubes as shown in Figure 4. The oxidation of divalent iron led to presence of Fe^2+^ and Fe^3+^ in addition to Ti^4+^ inside the nanorods that converted them to nanotubes because of the extreme Kirkendall effect [53].

### 3.5. Magnetic Properties

**Figure 11:**
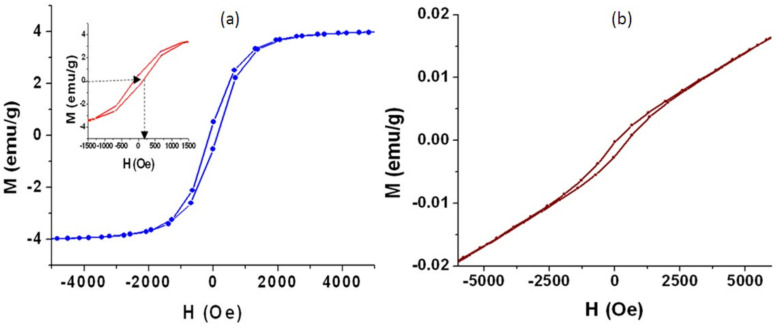
Magnetic properties of (**a**) TF-2 and (**b**) TF-3.

It is known that titanium dioxide is magnetically inactive. By combining with iron oxide, the magnetic behavior of titanium iron oxide became clear, as seen in the samples TF-2 and TF-3. The parameters of magnetization of the prepared nanotubes of the sample TF-2 were measured by Vibrating Sample Magnetometer at 300 K through applying magnetic field (H) between –5000 Oe and +5000 Oe. By plotting the magnetization (M) with the magnetic field (H), the curve of M–H of the sample TF-2 showed that the nanotubes of titanium iron oxide are magnetically active. With raising the external magnetic field of strength (H), the magnetization of TF-2 increased to reach saturation at 4 emu/g as seen in Figure 11a. Also, the magnetization and demagnetization processes showed that the sample TF-2 has a hysteresis loop. It means that the prepared nanotubes of titanium iron oxide belong to ferromagnetic materials.

By focusing on the low field region of the magnetic field, the inset of the Figure 11a indicated that the coercivity (Hc) of the sample TF-2, which determined from the reverse magnetic field used for demagnetizing the ferromagnetic material to be zero, is 110 emu/g. With returning the external magnetic field to equal zero, the remnant magnetization could be measured to be 1 emu/g indicating that not all domains of the nanotubes reverted to the original directions. This ferromagnetic behavior is due to the multi-domains of the titanium iron oxides of nanotubes that formed from aggregating nanoparticles. The alignment of atomic magnetic moments within the multi-domains interacted with the external magnetic field leading to clear hysteresis loop and saturation magnetization. These magnetic properties of sample TF-2 are important for re-using processes because it is easily separated in the optical applications by an external magnet.

By dividing remnant magnetization/saturation magnetization, the remanence ratio of the sample TF-2 is 0.25. It is known that the typical value of cubic anisotropy is 0.8 while the typical value of uniaxial anisotropy is 0.5. We can suggest that the anisotropy of the sample TF2 is predominantly uniaxial type because it is closer to the value 0.5. This uniaxial type of TF-2 could be explained according to the nano size of their nanotubes. Because of the nano size of the sample TF-2, surface effects become more pronounced. Therefore, the surface anisotropy can cause large deviation from the bulk behavior in the sample TF-2. At the surface, the spin has a nearest neighbor on one side and at the same time none on the other side, so that the exchange energy at the surface is not similar as in the bulk. This gives rise to surface anisotropy in the nanotubes. Because any surface energy should be a tendency of the surface spins to be either parallel or perpendicular to the surface, exchange coupling between surface and core atoms tends to create a unidirectional anisotropy. It means that the interaction between the surface and the core spins produces a uniaxial anisotropy of large magnitude although the fact that the bulk counterparts possess cubic anisotropy. 

In the case of the sample TF-3, the hysteresis loop became smaller indicating that the coercivity (H_c_) decreased to be 30 emu/g as seen in Figure 11b. In addition, there is no magnetic moment saturation observing in the sample TF-3. In the same trend, the remnant magnetization decreased to be 0.02 emu/g. These data indicated that the sample TF-3 has mixed phases behavior of ferromagnetic and antiferromagnetic cluster. The reason for the reduced coercivity may be due to existence of a significant fraction of superparamagnetic nanorods or the incomplete ordering and presence of other phases.

### 3.6. Optical Properties

**Figure 12:**
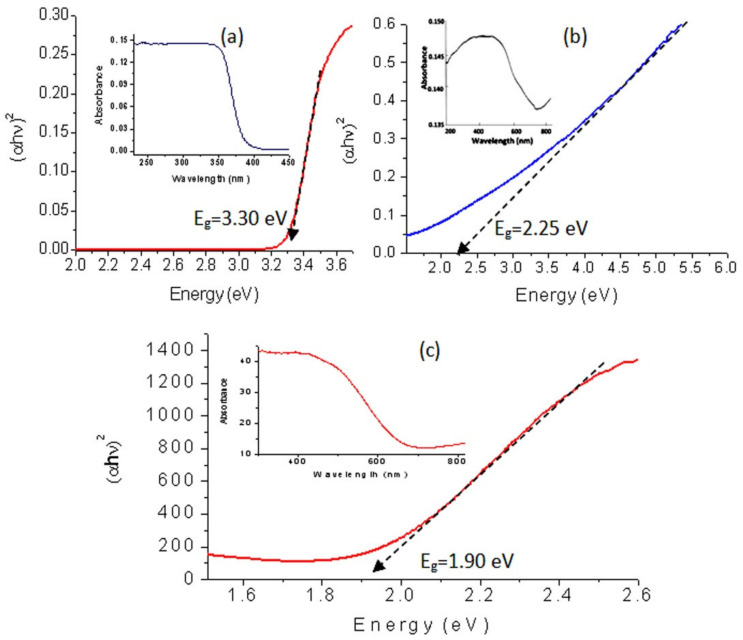
UV-Vis absorbance and band gap of (**a**) TTO-450, (**b**) TF-2 and (**c**) TF-3.

Titanium dioxide is considered to be one of the most famous photo-active materials in literature. There have been many attempts to modify the structure and the morphology of titanium dioxide to decrease its band gap. In this trend, the optical behavior of the titanium dioxide nanoparticles and their nanocomposites were studied by the UV-Vis absorption spectra that used a powerful tool for providing important details about their absorbance and band gaps. Figure 12a (inset) showed that the prepared nanoparticles of titanium dioxide are active in the UV region and inactive in the visible region because they have absorption in the range of wavelength 250–350 nm and there is no absorption in the range of wavelength 400–800 nm. The calculations of the band gap energy of the prepared titanium dioxide confirmed this data. Figure 12a, which was plotted by the relation between (αhν)^2^ and energy (hν) and determined the band gap energy E_g_ of the prepared TiO_2_ through extending the straight line to the (hν) axis to obtain the optical band gap energy at (αhν)^2^ of 0, showed a little wide band gap. The value of the band gap energy at room temperature was 3.30 eV agreeing with the electronic excitation from the valence band to the conduction band of conventional titanium dioxide. 

By converting the nanoparticles of TiO_2_ to nanotubes through their combination with di- and tri-valent iron, the optical properties improved as shown in Figure 12b,c. The absorbance of the sample TF-2 shifted to the visible region in addition to the UV region. A clear absorbance was observed in the range of wavelength 200–700 nm with maximum value at λ = 500 nm as seen in Figure 12b (inset). This improvement was confirmed by the calculations of band gap energy. Figure 12b showed that the band gap energy of the sample TF-2 is 2.25 eV. By comparing with titanium dioxide, strong narrowing for the band gap energy was observed from 3.30 eV to 2.25 eV. It means that TF-2 is more active than titanium dioxide as confirmed in the next sections. 

This speculation was confirmed by converting the nanoparticles of TiO_2_ to hollow nanorods after combining with trivalent iron. In the same trend, Figure 12c (inset) showed a clear shift for the absorbance of the sample TF-3 toward the visible region. It showed that the absorbance edge is near to λ = 600 nm. By calculating the band gap energy, this shift became clearer. Figure 12c showed that the band gap energy of TF-3 is 1.90 eV. By comparing with both TF-2 and titanium dioxide, more narrowing was observed for the band gap of TF-3 indicating that the hollow nanorods morphology has a strong positive effect on the optical properties.

Many authors have reported that the optical band gap energy of titanium dioxide is decreased by combining with iron [52,53,54,55,56]. According to the results of Guo et al. [56], they indicated that the band gap energy of titanium dioxide decreased to be 1.99 eV and 2.01 eV depending on the different ratios of both iron and titanium. Also, Mohammadi and Fray [57] reported that the combination between iron and titanium led to narrowing for the band gap energy of their oxides to be 1.95 eV. By the study of Tang et al. [58,59], a clear reduction was observed for the band gap energy to be 2.06 eV in case of titanium iron oxide with 10.5% of Ti. The reduction of the band gap energy of titanium dioxide after combining with iron can be explained according to the shallow trap sites. Agreeing with Singh et al., the combination of iron with titanium dioxide produces shallow trap sites between the valance band and conduction band leading to decreasing for the band gap energy [60]. Where, the sites of Ti in titanium dioxide are occupied by Fe atoms producing shallow traps. In the current study, the reduction of the band gap energy was stronger than the previous studies. It may be due to the hollow nanorods morphology of iron titanium oxides.

### 3.7. Photocatalytic Degradation of Dyes

**Figure 13:**
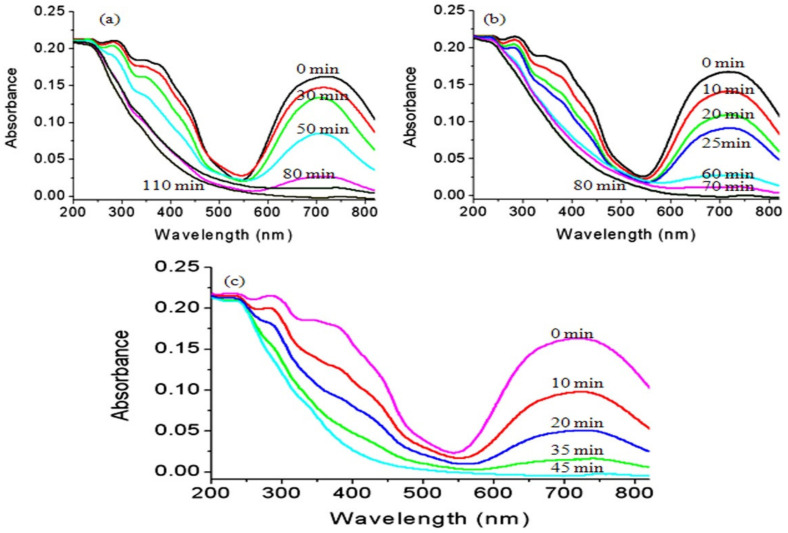
Photocatalytic degradation of Naphthol green B in presence of: (**a**) TTO-450, (**b**) TF-2 and (**c**) TF-3.

In the current study, the green dye of Naphthol green B was used as an example for colored pollutants. The photo activities of the prepared titanium dioxide and their nanocomposites with their different morphology were studied and used during photocatalytic degradation of Naphthol green B dye as photocatalysts. After irradiating the aqueous solution of the dye in presence of the UV light and one of the prepared photocatalysts, the absorbance of the liquid portion was measured to determine the concentration of Naphthol green B at a certain time. The degradation of the organic structure of Naphthol green B was determined by the reduction of its absorbance at λ_max_ of 714 nm as seen in Figure 13. Also, the absorbance of the organic phenyl groups of the dye at 320 nm and 400 nm gradually decreased with irradiation time of UV light.

According to the blank experiment, which was performed without catalyst, Naphthol green B concentration did not change by the irradiation of light indicating high stability against light. By changing the irradiation time of UV light, the photocatalytic degradation of Naphthol green B was followed and used as an indicator for measuring the photo activity of the prepared nanoparticles of titanium dioxide, TF-2 and TF-3. The obtained results are summarized in Figure 13. 

Figure 13a showed that the photocatalytic degradation of Naphthol green B under UV light in the presence of the nanoparticles of titanium dioxide increased with the increase in irradiation time. After 110 min of the UV irradiation time, a complete decolorization of Naphthol green B was observed indicating a full degradation for the dye. By using the nanotubes of titanium iron oxide, the color of Naphthol green B completely disappeared in a shorter time than that of the nanoparticles of titanium dioxide. Where, Figure 13b revealed that the decolorization process of Naphthol green B was achieved after 80 min of the UV irradiation time in presence of the sample TF-2. It means that the photocatalytic degradation of Naphthol green B became faster because of the conversion of nanoparticles to nanotubes and transformation of anatasa phase of titanium dioxide to maghemite-titanian structure. 

In case of using new morphology of maghemite-titanian as a photocatalyst, the hollow nanorods morphology of titanium iron oxide was more effective in the photocatalytic degradation of Naphthol green B. Where, the absorbance of Naphthol green B arrived to zero after 45 min of the UV irradiation time in presence of the sample TF-3. Figure 13c showed that the absorbance of Naphthol green B strongly decreased by increasing the time of the UV irradiation time during the first 20 min. While, during the second 20 min, the reduction of the absorbance of the dye became a little low as shown in Figure 13c. This behavior can be explained according to two processes adsorption and photocatalytic degradation. In the first 20 min, the removal of the dye was achieved by the two processes. In the second 20 min, the removal of Naphthol green B was accomplished by only photocatalytic process. It means that the hollow nanorods of titanium iron oxide have used UV light to accelerate the degradation of Naphthol green B in a faster way than the nanotubes of titanium iron oxide and the titanium dioxide nanoparticles. In another way, we have used commercial titanium dioxide which was provided by WAKO Company as a reference standard. The results of the commercial titanium dioxide showed that a complete photocatalytic degradation of Naphthol green B was achieved after 90 min of UV irradiation. It means that the performance of hollow nanorods was two times higher than that of the reference standard. This finding confirmed that the hollow nanorods have a useful and effective role for removing dyes. In addition, the high performance of the hollow nanorods was confirmed by the results of the kinetics study of these reactions.

## 4. Discussion

The kinetics of degradation and decolorization of Naphthol green B through photocatalysis process using the nanoparticles, nanotubes and hollow nanorods were calculated by the next Equation:

ln [C_o_/C] = k × t (3)

The rate reaction constant is k. The initial concentration of Naphthol green B, which was expressed by the absorbance at time equal zero, is coded as C_o_. The concentration of Naphthol green B at different times is coded as C. By plotting the irradiation time in minutes against ln(C_o_/C), the diagrams could be employed for kinetically determining the type of reactions. 

**Figure 14:**
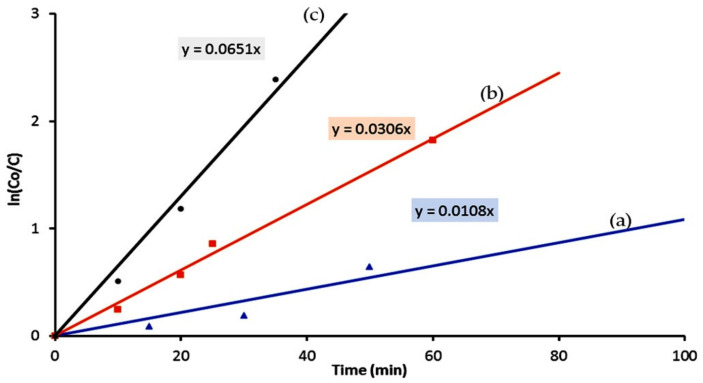
Kinetics study of the Photocatalytic degradation of Naphthol green B in presence of: (**a**) TTO-450, (**b**) TF-2 and (**c**) TF-3.

According to Figure 14, the diagrams indicated that the photocatalytic degradation and decolorization of Naphthol green B are pseudo-first-order reactions in case of using the titanium dioxide or their nanocomposites with different morphologies as photocatalysts.

Figure 14 showed that the rate reaction constant of the photocatalytic degradation of Naphthol green B in presence of the titanium dioxide nanoparticles is 0.011 min^−1^. By changing the nanoparticles to the nanotubes of titanium iron oxide, the reaction became faster because the rate reaction constant increased to be 0.031 min^−1^. Faster reaction was observed after using the hollow nanorods because the rate reaction constant of photocatalytic degradation of Naphthol green B was 0.065 min^−1^. The kinetics study concluded that the rate of photocatalytic degradation of Naphthol green B in presence of the hollow nanorods of titanium iron oxides increased to become six times higher than that of the nanoparticles of titanium dioxide. By comparing with the nanotubes of titanium iron oxides, the hollow nanorods showed a positive effect for the rate constant of photocatalytic reaction of Naphthol green B which was two times higher. The high performance of the hollow nanorods in the photocatalytic degradation reactions could be explained through the mechanism of the photocatalysis process. 

In the case of photo-active materials, there are three reactions for producing strong oxidizing agents which are used for catalytic degradation of dyes as shown in the next reactions:

Photocatalyst + UV light → electron^−^ (conduction band) + hole^+^ (valance band) (4)

Hole^+^ + H_2_O → H^+^ + • OH (free radical) (5)

Electron^−^ + O_2_ → • O_2_^−^ (6)

The Equation (4) considers the critical reaction in all processes. If the Equation (4) is fast, the pollutants will disappear in a short time. The wide band gap energy of titanium dioxide reduced the speed of the first reaction. Also, the nano size of the particles of titanium dioxide allowed the aggregation of electrons in the conduction band which accelerated the reverse reaction of Equation (4). It means that it accelerated a recombination reaction of electrons and holes. These two factors limited the performance of titanium dioxide for photocatalytic degradation of pollutants. In the case of the hollow nanorods, lowering and narrowing of the band gap energy accelerated the reaction (4) producing electrons and holes. At the same time, the morphology of the nanorods prevented the recombination reaction of electrons and holes through transporting the electrons to other places leading to easily separation between electrons and holes. Accordingly, it prevented the aggregation of electrons in the conduction band. It means that the nanorods will look like the wires in electric circuit.

## 5. Conclusions

In the current study, more than two objectives were achieved for developing titanium dioxide nanoparticles to be effective in the field of environmental applications. The first objective focused on fabrication of hollow nanorods and magnetic nanotubes of maghemite-titanian structures through Kirkendall process for the first time. These morphologies converted titanium dioxide to be magnetically active and easily separated from the solutions. Also, the optical properties of titanium dioxide improved after combing with iron and producing hollow nanorods and nanotubes through narrowing the band gap from 3.30 eV to 1.90 eV. Furthermore, this reduction of the band gap energy led to increasing the photocatalytic performance and activity of the hollow nanorods and the nanotubes comparing with the nanoparticles. This performance was proven by the decolorization and the mineralization of Naphthol green B at shorter time 45 min in case of using the hollow nanorods of the titanium iron oxides. These results were confirmed from the kinetic study which indicated that the reaction rate of the photocatalytic degradation of dyes in presence of the hollow nanorods was six times higher than that of the nanoparticles. Finally, it can be concluded that the combination between titanium and iron oxides through Kirkendall effect led to producing favorite and useful morphologies for environmental applications and inducing magnetic behavior for the photo-active materials in addition to improving their optical properties.

## Data Availability

Data available in a publicly accessible repository.
